# Synthesis of a Calcium Silicate Cement Containing a Calcinated Strontium Silicate Phase

**DOI:** 10.1155/2024/8875014

**Published:** 2024-01-25

**Authors:** Saeede Zadsirjan, Negar Parvaneh Dehkordi, Soolmaz Heidari, Farhood Najafi, Nazanin Zargar, Mojgan Feli, Sepideh Salimnezhad

**Affiliations:** ^1^Department of Endodontics, School of Dentistry, Shahid Beheshti University of Medical Sciences, Tehran, Iran; ^2^Department of Operative Dentistry, Dental Caries Prevention Research Center, Qazvin University of Medical Sciences, Qazvin, Iran; ^3^Department of Resin and Additives, Institute for Color Science and Technology, Tehran, Iran; ^4^Department of Endodontics, School of Dentistry, Qom University of Medical Sciences, Qom, Iran

## Abstract

**Objectives:**

The positive effects of strontium on dental and skeletal remineralization have been confirmed in the literature. This study aimed to assess the properties of a calcium silicate cement (CSC) containing a sintered strontium silicate phase.

**Materials and Methods:**

The calcium silicate and strontium silicate phases were synthesized by the sol–gel technique. Strontium silicate powder in 0 (CSC), 10 (CSC/10Sr), 20 (CSC/20Sr), and 30 (CSC/30Sr) weight percentages was mixed with calcium silicate powder. Calcium chloride was used in the liquid phase. X-ray diffraction (XRD) of specimens was conducted before and after hydration. The setting time and compressive strength were assessed at 1 and 7 days after setting. The set discs of the aforementioned groups were immersed in the simulated body fluid (SBF) for 1 and 7 days. The ion release profile was evaluated by inductively coupled plasma-optical emission spectrometry (ICP-OES). Biomineralization on the specimen surface was evaluated by scanning electron microscopy/energy dispersive X-ray spectroscopy (SEM/EDS). Data were analyzed by the Kolmogorov–Smirnov test, one-way and mixed ANOVA, Levene's test, and LSD post hoc test (*P*  < 0.05).

**Results:**

Except for an increasement in the peak intensity of hydrated specimens, XRD revealed no other difference in the crystalline phases of hydrated and nonhydrated specimens. The compressive strength was not significantly different at 1 and 7 days in any group (*P*  > 0.05). The setting time significantly decreased by an increase in percentage of strontium (*P*  < 0.05). Release of Ca and Si ions significantly decreased by an increase in percentage of strontium (*P*  < 0.05). SEM/EDS showed the formation of calcium phosphate deposits at 1 and 7 days.

**Conclusion:**

Incorporation of 10−30 wt% sintered strontium silicate phase as premixed in CSC can significantly decrease the setting time without compromising the compressive strength or biomineralization process of the cement.

## 1. Introduction

Cements with hydraulic tricalcium silicate base such as mineral trioxide aggregate (MTA) are biocompatible materials that have the potential to form hydroxyapatite and remineralize the hard tissue following contact with body fluids. They can affect the pulp cells and lead to gene expression and release of growth factors, and subsequently induce dentin formation. Also, their role in synthesis of root cementum and regeneration of periodontal ligament has been well-documented [1]. Such cements have antibacterial properties and optimal sealing ability. After setting, they show insignificant water solubility, and have optimal dimensional stability [1, 2]. Despite such favorable properties, they have drawbacks such as poor handling, long setting time [3, 4], poor mechanical properties, and decreased physical properties following reaction with body fluids [5].

Several methods have been used to enhance the properties of dental and bone cements. Addition of elements such as zinc, titanium, and strontium to calcium silicate ceramics has been suggested since they can affect the properties of these cements [6–8]. Of different elements, strontium can play a specific role in remodeling. Strontium ions positively enhance the activity of bone-forming osteoblasts and decrease the bone-resorption activity of osteoclasts. These ions are used for treatment of conditions such as osteoporosis where the balance between bone formation and resorption is deranged. Also, incorporation of strontium in bone and dental cements can induce the differentiation of mesenchymal stem cells in bone and teeth [9]. The bioavailability of orally taken strontium ions is only 20% [10]. Thus, local release of strontium ions at the bone defect site is preferred compared with increasing the oral dosage of strontium. Despite the abovementioned advantages, the results regarding the effects of addition of strontium to calcium silicate cements (CSCs) on other cement properties are controversial. No et al. [11] showed that increasing the percentage of strontium may increase bioactivity but can also adversely affect the mechanical properties of the cement. Huang et al. [12] reported longer setting time by increasing the percentage of strontium. Another property of strontium, compared with calcium, is its higher weight, which can cause greater radiopacity. Bismuth oxide, which is added to dental CSCs to confer radiopacity, can cause tooth discoloration. Thus, using strontium-containing compounds to achieve higher radiopacity with minimal tooth discoloration has been considered [13, 14].

This study aimed to describe the synthesis and characterization of a CSC containing tristrontium silicate. By conduction of different tests, this study aimed to answer the question whether addition of strontium can improve the properties of CSCs. It should be noted that strontium silicate was used as a source of strontium in premixed form in the present study. This method is different from the substitution of strontium ions in the structure of CSCs [15].

## 2. Materials and Methods

### 2.1. Synthesis of Calcium Silicate Cements

#### 2.1.1. Synthesis of Calcium Silicate Powder

The powder was synthesized by the sol–gel technique according to a study by Moon et al. [16]. Tetraethyl orthosilicate (TEOS) was used as a source of silicon, and calcium nitrate tetrahydrate was used as a source of calcium. To synthesize tricalcium silicate (C3S) and dicalcium silicate (C2S), 0.3 and 0.2 mol calcium nitrate tetrahydrate were used, respectively. The amount of TEOS in the synthesis of both materials was adjusted to 0.1 mol. The primary ingredients were dissolved in a solution of 70% ethanol, 5% polyethylene glycol (Mw 10,000), and 1% of 1 M HCl and stirred on a stirrer at 60°C for 3 hr. After gelation and drying, the obtained mixture was calcinated at 500°C and 1,200°C for 1 and 3 hr, respectively. For C3S, heat treatment was continued for another 8 hr at 1,450°C. The obtained material was ground and sieved using a 45 *µ*m sieve. The obtained powders were mixed with the ratio of C2S : C3S 30 : 70, and 5 wt% zirconium oxide and barium sulfate (for radiopacity) and 2% polyvinylpyrrolidone (thickener) were added to them and ground.

### 2.2. Synthesis of Strontium Silicate Powder ((SrO)_3_SiO_2_)

To synthesize tristrontium silicate by the sol–gel technique, 10.4 g of TEOS was mixed with 50 mL of water. The pH of the solution was adjusted at three by using 0.1 N nitric acid solution. After 30 min of stirring at room temperature, 22.4 g of strontium carbonate was added to the solution and stirred for 2 hr at 90°C on a heater stirrer. The contents of the beaker were placed in an oven at 120°C for 2 hr. The obtained powder was ground with a ball mill for 30 min, and then calcinated at 1,400°C for 4 hr. The obtained material was ground and sieved by a 45-*µ*m sieve.

### 2.3. Mixing of Calcium Silicate and Tristrontium Silicate Powders

Tristrontium silicate powder was mixed with calcium silicate powder by a laboratory mixer (Vortex, USA) with 10, 20, and 30 wt% concentrations. Then, the mixed powders were ground and used as tristrontium silicate-containing groups in the next steps.

### 2.4. Synthesis of the Liquid Phase of the Cement

The liquid phase of the cement in all groups included a mixture of 15% calcium chloride with 5% polycarboxylate super plasticizer and 80% water. All materials used in the present study were purchased from Sigma Aldrich (Gillingham, UK) and Merck KGaA (Darmstadt, Germany).

The study groups were as follows:

1. CSC as the control group (CSC)
2. CSC containing 10% strontium silicate (CSC/10Sr)
3. CSC containing 20% strontium silicate (CSC/20Sr)
4. CSC containing 30% strontium silicate (CSC/30Sr)

### 2.5. X-Ray Diffraction (XRD)

XRD (X′Pert PROMPD, PANalytical, Netherland) was conducted for the synthesized powders and also the cements after setting for characterization of the crystalline phases (40 kV, 40 mA, step size: 0.02, range: 5°−120°2*ϴ*).

### 2.6. Assessment of Particle Size

The particle size was measured by wet-sizing using Sympatec HELOS and CUVETTE laser diffraction system (Sympatec GmbH, Clausthal-Zellerfeld, Germany) with a R3 lens (0.9–175 *μ*m).

### 2.7. Measurement of the Compressive Strength of CSCs

ISO-9917-1 was used for preparation of specimens for the compressive strength test [17]. The powder and liquid of all four cements were mixed in appropriate ratios on a glass slab with a metal spatula. The mixing ratios were different for each group. The powder-to-liquid ratio was 2 : 1 (6 mg of powder with 1 mL of liquid) in the strontium-free and CSC/10Sr groups, and 2 : 2 (6 mg of powder with 2 mL of liquid) in the CSC/20 Sr and CSC/30 Sr groups to achieve optimal consistency for handling. After mixing, the materials were well-packed in cylindrical molds with 4-mm diameter and 6-mm height to ensure no void formation. The molds were then incubated at 37°C and >90% moisture for 24 hr and 7 days (*n* = 6). The fracture strength of specimens was measured by a universal testing machine (Z020; Zwick Roell, Ulm, Germany) by applying 2,000 kg vertical load at a crosshead speed of 0.5 mm/min. The 4 p/*πd*^2^ formula was used to measure the compressive strength, where *p* is the maximum recorded force in newtons (N) and *d* is the diameter of the sample in millimeters (mm).

### 2.8. Setting Time Assessment

The cement specimens were prepared as explained for the compressive strength test. The cements were packed in disc-shaped molds with 10-mm diameter and 2-mm height (*n* = 10). The specimens were then incubated at 37°C and 90% humidity. The setting time was measured by a Gillmore needle by applying 456 ± 0.5 g load with a needle diameter of 1 ± 0.1 mm. For this purpose, the indenter was lightly pressed over the material surface every 5 min for a total duration of 5 s. This process was repeated until no indentation remained on the surface.

### 2.9. Ion Release Profile, pH Alterations, Weight Loss, and Biomineralization on the Surface of CSCs Following Immersion in Simulated Body Fluid (SBF)

Disc-shaped specimens (*n* = 3) with 2-mm thickness and 10-mm diameter were fabricated and incubated at 37°C and 90% humidity for 7 days. The specimens were then weighed, and immersed in containers containing SBF with 0.01 g/mL ratio according to the technique described by Kokubo and Takadama [18]. The containers containing the specimens were incubated at 37°C for 1 and 7 days. The pH of the SBF with the specimens immersed in it was measured after 1, 3, and 7 days by a pH meter (Jenway3310, England). After 1 and 7 days, the disc-shaped specimens were removed and the solution was filtered by 0.22 *µ*m filter paper. The specimens were then diluted in nitric acid solution in 1 : 10 and 1 : 100 ratios. The ion release profile was assessed by inductively coupled plasma-optical emission spectrometry (ICP-OES; Varian, Vista-Pro model, Australia). The specimens were weighed again after 1 and 7 days in ambient air after drying. The percentage of weight loss was calculated using the following formula:

(1) $$\begin{array}{c} \frac{\text{Secondary weight}-\text{primary weight}}{\text{Primary weight}}\times 100. \end{array}$$

Scanning electron microscopy/energy dispersive X-ray spectroscopy/ (SEM/EDS) was conducted to analyze the potential of CSCs to form calcium phosphate precipitations after immersion in SBF. For this purpose, 7-day discs that were used for the ion release test were subjected to SEM/EDS (TESCAN MIRA XMU, Czech Republic; Acceleration, Voltage: 20 kV) assessment. For further clarity of images, the specimens were gold-coated.

### 2.10. Statistical Analysis

Normal distribution of data was evaluated by the Kolmogorov–Smirnov test, and homogeneity of variances was assessed by the Levene's test. Repeated measures mixed ANOVA and one-way ANOVA were applied to compare the results of different groups at different time points. Pairwise comparisons of the groups were carried out by the LSD post hoc test. *P*  < 0.05 was considered statistically significant.

## 3. Results

### 3.1. XRD

**Figure 1:**
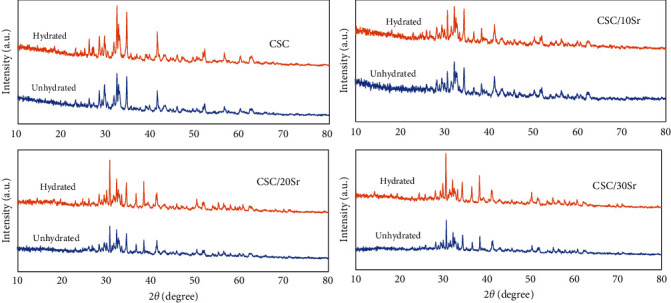
XRD analysis of the hydrated and nonhydrated specimens showed a similar pattern.

**Figure 2:**
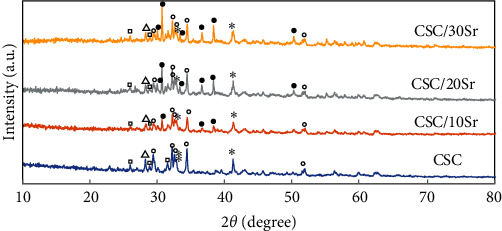
XRD analysis of the study groups showing the formation of respective crystalline phases. Open circle, tricalcium silicate (Ca_3_SiO_5_); astrik, dicalcium silicate (Ca_2_SiO_4_); solid circles, tristrontium silicate (Sr_3_SiO_5_); open triangles, zirconium oxide (ZrO_2_); open squares, barium sulfate (BaSO_4_).

A similar pattern with respect to crystalline phases was noted when comparing hydrated and nonhydrated specimens, with the difference that the peaks of the crystalline phases had a lower intensity in the nonhydrated specimens (Figure 1). The main phases in all four hydrated cements were tricalcium silicate and dicalcium silicate. In groups containing strontium, a crystalline phase of strontium silicate was also identified in addition to the abovementioned two phases. The peaks related to dicalcium silicate and tricalcium silicate were identified at around 32.2°–34.4° (2*ϴ*) and also 41.2°. Tristrontium silicate was mainly detected at around 36.5°, 38.2°, and 30.6° (2*ϴ*). The peaks related to zirconium oxide and barium sulfate were also identified (Figure 2).

### 3.2. Particle Size

The particle size of each group is reported in Table 1. No significant difference existed in particle size among the three groups (*P*  > 0.05).

### 3.3. Setting Time

The mean setting time in the CSC group was significantly longer than that in other three groups (*P*  < 0.05). The CSC/30Sr group had a significantly shorter setting time than the other groups (Table 2).

### 3.4. Compressive Strength

The compressive strength significantly increased at 7 days compared with Day 1 in all groups (*P*  < 0.001). No significant difference existed in the mean compressive strength at different time points among the study groups (Table 2).

### 3.5. Ion Release Profile, pH Alterations, and Weight Loss

**Figure 3:**
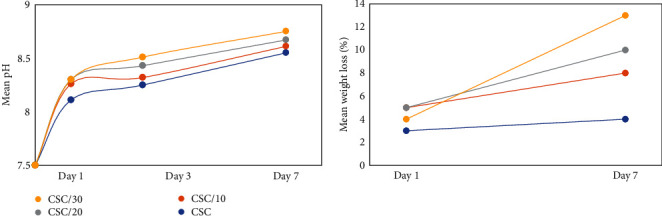
Comparison of the mean pH and solubility of different groups.

The effect of time on the release of Ca, Si, and Sr ions was significant at the two time points, indicating a significant increase in ion release at 7 days compared with 1 day. The effect of group on the ion release profile was also significant (*P*  < 0.001), indicating presence of a significant difference in the mean amount of released ions among the study groups (*P*  < 0.001). The mean amount of released Ca at both 1 and 7 days significantly decreased by an increase in the amount of strontium silicate. The mean amount of released Si ions at Day 1 was significantly the highest and the lowest in CSC and CSC/30Sr groups, respectively. At 7 days, the release of Si ions significantly decreased by an increase in the amount of strontium silicate. At both time points, the release of Sr ions significantly increased by an increase in the amount of strontium silicate (Table 3). Assessment of pH changes revealed an increase in pH in all groups over time, and by an increase in the amount of strontium, the solution became more alkaline. The trend of change in weight loss was almost similar to that of pH, such that the weight loss was the lowest in the CSC and the highest in the CSC/30Sr group (Figure 3).

### 3.6. SEM/EDS

**Figure 4:**
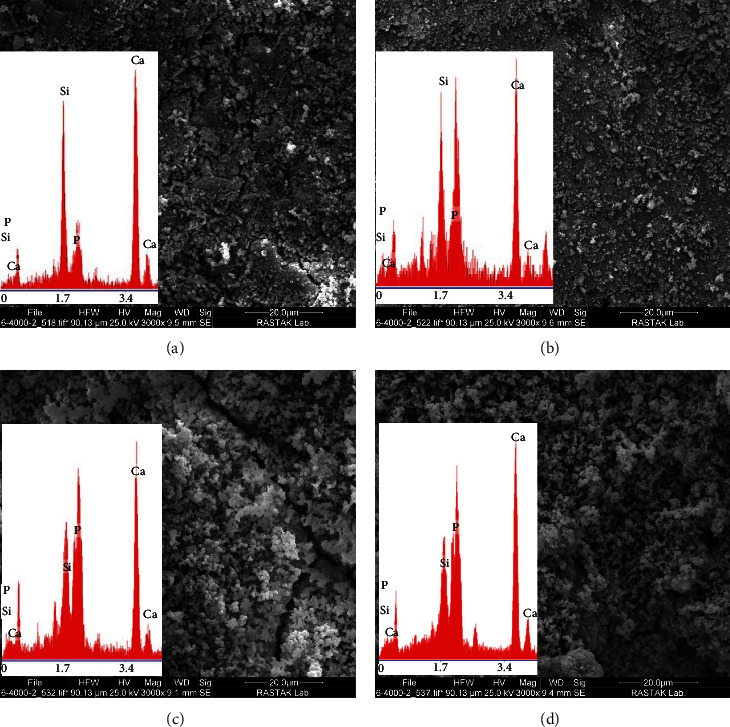
SEM/EDS analysis of disc-shaped specimens of (a) CSC, (b) CSC/10Sr, (c) CSC/20Sr, and (d) CSC/30Sr immersed in SBF for 1 day at ×3,000 magnification.

**Figure 5:**
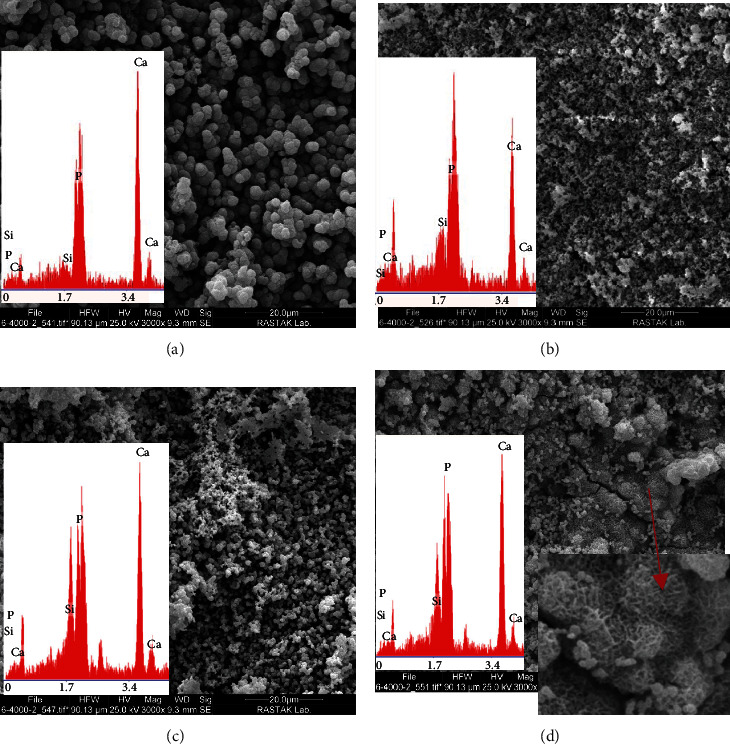
SEM/EDS analysis of disc-shaped specimens of (a) CSC, (b) CSC/10Sr, (c) CSC/20Sr, and (d) CSC/30Sr immersed in SBF for 7 days at ×3,000 magnification.

Figures 4 and 5 show the SEM/EDS images of specimens following immersion in SBF for 7 days. In all groups, calcium phosphate precipitations were noted at 1 and 7 days. According to the SEM/EDS analysis, the amount of formed calcium phosphate precipitations at 7 days was greater than that at 1 day.

## 4. Discussion

According to the XRD results (Figure 2), the main phases of unset bioceramic cements were tricalcium and dicalcium silicate. Identification of the precise location of peaks related to dicalcium silicate is difficult due to overlapping with the tricalcium silicate peaks. The peak related to tristrontium silicate was noted in groups containing it. By an increase in the amount of strontium, the intensity of this peak increased, and the intensity of tricalcium silicate peak decreased. Appearance of a separate peak for tristrontium silicate indicates physical mixing of silicates for the synthesis of strontium-containing calcium silicate powder. The hydrated and nonhydrated specimens showed a similar pattern of peaks with the difference that peaks had a higher intensity in the hydrated specimens (Figure 1). This result was in agreement with some previous observations [19, 20]. Hydrated calcium silicate and calcium hydroxide are the main products of the hydration reaction of CSCs. In the setting reaction, tricalcium silicate and dicalcium silicate form hydrated calcium silicate gel following reaction with water. Due to its amorphous nature, it cannot be easily detected by an XRD analysis. A similar hydration reaction occurs for strontium silicate. The crystallin form of calcium hydroxide can be detected by XRD analysis [21]. Nonetheless, absence of a specific phase of calcium hydroxide in XRD of some commercial MTA products (similar to the present work) may reflect a defect in cement hydration. A number of factors such as the size and morphology of the particles and their chemical composition can play a role in this regard. It has been demonstrated that smaller particles and calcium sulfate can affect the setting process, and result in appearance of calcium hydroxide peak in XRD [22]. In another study, no trace of crystalline calcium hydroxide was attributed to subsaturated concentrations of the soluble calcium hydroxide [21].

In the present study, the CSC/30Sr group showed the shortest setting time (Table 2). The setting time depends on the particle size, powder/liquid ratio, and chemical composition of material [1, 3, 23]. In the present study, the particle size was almost the same in all specimens. Thus, the difference between the setting time of the groups with and without tristrontium silicate may be attributed to the presence of tristrontium silicate or difference in powder/liquid ratio when mixing. In hydraulic CSCs, dicalcium silicate and tricalcium silicate have hydraulic properties, and react with water or aqueous solutions to form calcium silicate hydrate (C–S–H) as the main hydration product. It participates in the setting reaction of materials, and at the same time, increases their strength [3, 4]. The strontium ion is similar to the calcium ion with respect to the size of atomic radius and electrical charge. Thus, strontium silicate can similarly react with water. Some studies have shown that strontium in strontium dicalcium silicate cements may interfere with the formation of hydrated calcium silicate gel and prolong the primary setting time [12, 24]. It should be noted that in the aforementioned studies, the strontium ion was incorporated in the structure of calcium silicate material by substitution, which is different from the synthesis method used in the present study where strontium was used in strontium silicate in premixed form. Difference in setting times can be due to the application of different amounts of liquid for optimal handling. In the present study, a higher powder-to-liquid ratio was used in groups without tristrontium silicate and with 10% tristrontium silicate, compared with groups containing 20% and 30% tristrontium silicate. The liquid phase of the cement also contains calcium chloride, which may be another reason for the difference in setting time among the groups. The efficacy of calcium chloride in reduction of setting time of MTA has been previously reported [25].

The mean compressive strength values obtained for the cements in the present study are similar to some, and different from some other values reported in the literature for calcium silicate compounds (Table 2). Variations in the results can be attributed to the size of specimens, powder composition, preparation technique, and powder to liquid ratio. For instance, lower amounts of liquid yield higher strength [26–28]. The present results showed that in all groups, the compressive strength at 7 days was significantly higher than that at 24 hr. The compressive strength of cements is also related to their setting time. It appears that as the time passes since setting, the compressive strength further improves. Since the hydration speed of dicalcium silicate is slower than that of tricalcium silicate, it later participates in a reaction. Thus, the compressive strength of CSCs is maximized a couple of days after mixing, upon completion of the setting reactions [4]. In the present study, the difference among the groups was not significant in compressive strength. Nonetheless, specimens containing tristrontium silicate showed lower compressive strength after 7 days. Change in compressive strength can be attributed to changes in the crystalline structure by addition of different amounts of tristrontium silicate as well. Despite the high similarity of calcium and strontium ions, the ionic radius of strontium ions is slightly larger than that of calcium ions, which can decrease the connectivity of the silicate network, and decrease the compressive strength of the material [29].

In ceramic materials, the relationship of density and strength should be taken into account, such that by a reduction in density, the strength often decreases. Strontium ions can affect the sintering kinetics and condensation, and subsequently the mechanical properties of ceramics [11]. The effect of addition of strontium silicate on the microstructure of silicate cements should be assessed more comprehensively. In the present study, the reduction in compressive strength following addition of tristrontium silicate can be attributed to the use of higher amounts of calcium chloride. The results of studies regarding the effect of calcium chloride on compressive strength are controversial [30, 31].

The calcium silicate group devoid of strontium silicate (CSC) released higher amounts of calcium into the SBF compared with the calcium silicate groups modified with tristrontium silicate (Table 3). Calcium release decreases by a reduction in the amount of calcium silicate in the cement composition. The concentration of strontium depends on the composition of the modified cement. The higher the amount of strontium, the higher the concentration of strontium ions in SBF would be. In the present study, the calcium ion release can be partly attributed to addition of calcium chloride [32]. The release of Si ions was low in all study groups. In order to explain this finding, the mechanism of formation of hydroxyapatite in the body or simulated environments should be taken into account. In the setting reaction of cements, depending on the type of cement, hydrated calcium silicate, hydrated strontium silicate, calcium hydroxide, or strontium hydroxide may be formed. According to Gandolfi et al. [33], the surface of cement specimens is initially smooth following immersion in phosphate-buffered saline. Gradually, the cement surface porosities are coated with SiOH gel. After 5–24 hr, the cement surface is coated with spherical hydroxyapatite crystals, and over time, the number of hydroxyapatite layers increases until 28 days [33]. Thus, it appears that at 1 and 7 days, Si ions are mainly bound to oxygen in the structure of silicate or bound to OH. In calcium silicate and strontium-containing calcium silicate groups, similar amounts of silicate and subsequently Si ions are present. However, lower release of Si ions in strontium-containing groups can be due to two reasons. Strontium ions may prevent movement and release of Si due to large atomic radius [15]. Nonetheless, some studies showed that slightly larger ionic radius of strontium compared with calcium may change the crystalline network and cause its degradation and subsequently increased solubility of the silicate structure and ion release [34]. Moreover, the solubility of strontium hydroxide is higher than that of calcium hydroxide [35] (both products form following cement hydration), which can lead to greater dissolution of cement and greater ion release; however, this statement is in contrast to the present findings. A suggested hypothesis is that the ion release was assessed at 1 and 7 days in the present study. Thus, Si ion release in strontium-containing groups could have been similar or even higher than that in calcium silicate group in early hours. Subsequently, the released ions faster enter the reaction with SBF and form SiOH, which can serve as a core for nucleation of hydroxyapatite [3], and eventually result in lower identification of Si ions in the ICP analysis. The ICP analysis should be conducted in early hours to more precisely address this topic.

The hydration reaction of CSC results in formation of calcium hydroxide. Release of calcium and hydroxyl ions increases the pH. Similarly, formation of strontium hydroxide, which is a strongly alkaline compound, increases the pH. High pH decreases the solubility of hydroxyapatite and enhances its formation [36] Although the mean pH of the synthesized materials was close to that of ProRoot MTA (Maillefer, Dentsply, Switzerland), it was still lower than the pH of many CSCs (Figure 3). In the present study, the materials were immersed in SBF 1 week after complete setting, which is different from the immersion of fresh material or shortly after setting. Also, type of solution in which the materials are immersed may vary [3]. In the CSC/20Sr and CSC/30Sr groups, higher amounts of calcium chloride in the solution were used. The results regarding the effects of calcium chloride on pH are controversial. Increase in pH of cement for up to 72 hr and subsequent reduction in pH after 7 and 14 days have been attributed to the presence of calcium chloride (with a pH of 4.4). Some others also reported an increase or reduction in pH of CSC following the use of calcium chloride [25, 32, 37]. Some other reasons for higher pH in strontium silicate groups can be the formation of strontium hydroxide, which has higher solubility and alkalinity than calcium hydroxide, or less integrated structure of strontium silicate [35, 38]. The alteration pattern of solubility matched the pH alterations in the present study (Figure 3). The present results showed increased solubility at 7 days compared with 1 day in all groups. The modified cements showed higher solubility than the group devoid of tristrontium silicate. According to ISO 6876, the optimal solubility of endodontic cements is lower than 3% [39]. Solubility and release of calcium ions are among the necessary parameters to achieve bioactivity [40]. Nonetheless, high solubility can decrease sealing ability. It should be noted that according to Parirokh and Torabinejad [4], although most studies measure solubility by calculating the difference between the primary and secondary weight, this method basically shows the washout of water-soluble components of the cement rather than its solubility, because in this technique, the material may absorb water or parts of it may become separated.

As shown on SEM micrographs (Figures 4 and 5), globular-shaped deposits were formed on the surface of all specimens after 1 and 7 days, showing calcium and phosphate peaks in SEM/EDS analysis. The formation of calcium phosphate precipitations was lower on Day 1. Thus, the peak related to Si, indicating the presence of silicate in the cement structure, had a higher intensity in SEM/EDS analysis. The thickness of precipitations increased gradually until Day 7, such that the peak related to Si at Day 7 had a much lower intensity while strong calcium and phosphate peaks were observed. The CSC and CSC/10Sr groups had lower amounts of calcium phosphate precipitations on Day 1 and had a stronger Si peaks. This finding may indicate slower reaction pace with SBF in these two groups. At Day 7, the surface of all cement specimens was coated with high amounts of calcium phosphate precipitations. In the CSC/30Sr group, flake-like precipitations along with strong calcium and phosphate peaks may indicate the formation of hydroxyapatite crystals. Although, the globular appearance may also be related to the formation of hydroxyapatite, precise timing of maturation of calcium phosphate precipitations and their conversion to hydroxyapatite after setting should be further assessed by XRD analysis.

## 5. Conclusion

Increasing the amount of strontium silicate as the second phase of CSC (from 10 to 30 wt%) significantly decreased the setting time. Although, addition of this phase decreased the compressive strength, this reduction was not significant. All cements optimally reacted with SBF, and showed the formation of calcium phosphate phase. Considering the confirmed effects of strontium on biomineralization, it appears that use of biphasic CSC (along with strontium silicate) should be taken into account. Future cellular and molecular biological analyses are required for further assessment of such biphasic cements.

## Figures and Tables

**Table 1:** Particle size of synthesized powders in different groups.

Mean particle size (*μ*m) materials	X90 ^*∗∗*^	X50 ^*∗*^
CSC	32.38	11.49
CSC/10Sr	33.00	12.11
CSC/20Sr	31.05	11.71
CSC/30Sr	35.65	15.01

^*∗*^X50: mean particle size of 50% of the material is lower than the mentioned value.  ^*∗∗*^X90: mean particle size of 90% of the material is lower than the mentioned value. No significant difference was noted.

**Table 2:** Comparison of compressive strength (MPa) and setting time (min) of different groups.

Groups	Compressive strength (mean ± std. deviation)		Setting time (mean ± std. deviation)
	Day 1	Day 7	
CSC	6.91 ± 3.02^a^	20.29 ± 8.01^b^	138.50 ± 12.48^A^
CSC/10Sr	5.01 ± 2.31^a^	13.17 ± 3.27^b^	127.50 ± 6.77^B^
CSC/20Sr	6.46 ± 3.54^a^	14.73 ± 7.49^b^	129.20 ± 9.70^B^
CSC/30Sr	5.71 ± 2.19^a^	16.77 ± 7.07^b^	95/00 ± 9.13^C^

Different letters indicate presence of a significant difference.

**Table 3:** Comparison of Sr, Ca, and Si ion release (ppm) in different groups.

Ion release (mean ± std. deviation)		CSC	CSC/10Sr	CSC/20Sr	CSC/30Sr
Ca	Day 1	311.67 ± 2.52^a^	272.67 ± 6.43^b^	253.67 ± 2.52^b^	230.00 ± 2.64^c^
	Day 7	505.00 ± 5.58^d^	416.67 ± 4.04^e^	327.33 ± 2.87^f^	321.00 ± 3.00^g^

Si	Day 1	7.77 ± 0.22^a^	4.41 ± 0.12^b^	4.14 ± 0.12^b^	2.88 ± 0.08^c^
	Day 7	21.05 ± 0.60^d^	19.36 ± 0.57^e^	13.09 ± 0.37^f^	8.66 ± 0.27^g^

Sr	Day 1	—	205.00 ± 3.60^a^	354.67 ± 5.13^b^	434.33 ± 6.03^c^
	Day 7	—	366.67 ± 5.51^d^	555.33 ± 8.50^e^	738.00 ± 9.54^f^

Different letters indicate presence of a significant difference. Pairwise comparisons were conducted independently for each ion.

## Data Availability

The data used to support the findings of this study were supplied by the corresponding author under license and data will be available on request. Requests to access these data should be made to the corresponding author.
